# On the Effectiveness of Oxygen Plasma and Alkali Surface Treatments to Modify the Properties of Polylactic Acid Scaffolds

**DOI:** 10.3390/polym13101643

**Published:** 2021-05-18

**Authors:** Ricardo Donate, María Elena Alemán-Domínguez, Mario Monzón

**Affiliations:** Grupo de Investigación en Fabricación Integrada y Avanzada, Departamento de Ingeniería Mecánica, Universidad de Las Palmas de Gran Canaria, Campus Universitario de Tafira s/n, 35017 Las Palmas, Spain; mariaelena.aleman@ulpgc.es (M.E.A.-D.); mario.monzon@ulpgc.es (M.M.)

**Keywords:** polymer, low-pressure plasma, plasma treatment, surface modification, biomedical applications, additive manufacturing, toluidine blue method, enzymatic degradation

## Abstract

Surface modification of 3D-printed PLA structures is a major issue in terms of increasing the biofunctionality and expanding the tissue engineering applications of these parts. In this paper, different exposure times were used for low-pressure oxygen plasma applied to PLA 3D-printed scaffolds. Alkali surface treatments were also evaluated, aiming to compare the modifications introduced on the surface properties by each strategy. Surface-treated samples were characterized through the quantification of carboxyl groups, energy-dispersive X-ray spectroscopy, water contact angle measurements, and differential scanning calorimetry analysis. The change in the surface properties was studied over a two-week period. In addition, an enzymatic degradation analysis was carried out to evaluate the effect of the surface treatments on the degradation profile of the 3D structures. The physicochemical characterization results suggest different mechanism pathways for each type of treatment. Alkali-treated scaffolds showed a higher concentration of carboxyl groups on their surface, which enhanced the enzymatic degradation rate, but were also proven to be more aggressive towards 3D-printed structures. In contrast, the application of the plasma treatments led to an increased hydrophilicity of the PLA surface without affecting the bulk properties. However, the changes on the properties were less steady over time.

## 1. Introduction

The use of 3D scaffolds constitutes one of the most promising approaches in the biomedical field to regenerate damaged tissue. As scaffolds act as artificial structures that support and direct new tissue formation, various requirements must be fulfilled, including biocompatibility, suitable mechanical properties, interconnected porosity, promotion of cell’s attachment and growth, supported vascularization, and ease of sterilization [1]. Unlike other commonly used biomaterials in the tissue engineering (TE) field, such as titanium [2] and bioceramic materials [3], polymeric scaffolds also possess a distinctive feature: their biodegradability. As the byproducts of the polymer’s degradation process are excreted through the usual metabolic pathways, a complete integration of the scaffold can be achieved, avoiding the need for an explant surgical procedure [4]. Conversely, the degradation profile of polymeric scaffolds needs to be carefully adjusted to ensure sufficient mechanical support during new tissue formation and that no immune or inflammatory response happens in the implantation site due to the release of acidic byproducts [5].

Polylactic acid (PLA) is a biodegradable thermoplastic aliphatic polyester that is used extensively for scaffold manufacturing. Apart from its biocompatibility, adjustable biodegradability, and suitable mechanical properties, PLA has good processability by Additive Manufacturing (AM) techniques (ISO/ASTM 52900:2015), allowing for the development of patient-specific constructs [6]. The use of AM technologies for biopolymer manufacturing and biological and medical applications gained interest over the last decades [7] mainly due to the great control that they offer in terms of pore size, pore shape, and porosity of the structures [8,9]. On the other hand, the main drawbacks for the biomedical application of PLA scaffolds include (a) release of acidic degradation byproducts; (b) poor toughness; (c) lack of reactive side chain groups, which limits the treatments available for improving its surface properties, and (d) low hydrophilicity, which hinders cell attachment and scaffold–tissue interaction.

To overcome the limitations of PLA, different surface treatments can be applied to the scaffold to modify its topography or surface chemistry, including plasma deposition, plasma sputtering and etching, physical entrapment of small functional molecules, aminolysis, and hydrolysis [10,11]. Among them, one simple and effective strategy is the alkali treatment, based on the immersion of the PLA scaffolds in sodium hydroxide (NaOH) solutions. The hydrolysis of the ester bonds of PLA occurs due to the nucleophilic attack of hydroxide ions on the carbonyl carbon [12,13], leading to the incorporation of hydroxyl (-OH) groups and the increase of surface roughness; thus, increasing the hydrophilicity of the base material [14,15]. Generally, after alkali hydrolysis, the samples are washed with inorganic acids to remove the excess of NaOH. However, if organic acids are used instead, the carboxyl groups (–COOH) of these compounds can hydrolyze the PLA’s ester bonds [14] while also removing the excess of NaOH. Carboxyl groups incorporated into the PLA surface lead to an enhancement of roughness and wettability [14,16], serving as anchoring points for biological substances [17,18] and promoting the cell adhesion and proliferation processes [19,20]. The modifications generated by alkali treatment methods depend strongly on the concentration of the solution used, and the treatment time could affect the surface morphology and mechanical properties of the scaffolds [21].

In contrast, plasma treatments allow for the modification of the polymeric surface without affecting the bulk properties. The effects generated on the surface depend on the working conditions (power/voltage and time of exposure) and the carrier gas used. In the case of oxygen plasma treatments, hydroxyl and carboxyl groups can be incorporated into the surface of PLA scaffolds [22,23], achieving a reduction of the water contact angle while affecting the roughness of the samples at a nanometric scale [23,24]. Overall, the plasma treatment of PLA samples can lead to an enhanced cell adhesion, morphology, and proliferation [23,25,26]. The change on the chemical structure of the surface material may also have an impact on the bulk crystallinity of 3D-printed structures, as the width of the struts is low enough to be affected by the changes on the surface. While in some cases the modifications introduced by these surface treatments are enough to fulfill the requirements for the tissue-engineered PLA scaffolds [25], these treatments are usually combined with a later coating step based on the immobilization of bioactive substances within the polymeric matrix [18,27]. A subsequent coating procedure or the direct application of the surface-treated scaffolds should not be delayed too long, as the modifications generated by these treatments are not permanent, showing a progressive decay of the modifications generated on the polymer´s surface [28,29,30].

In this work, a comparison between alkali and plasma treatments of PLA scaffolds obtained by AM was carried out. These methods are broadly considered simple and cost-effective strategies to improve the biofunctionality of polymeric surfaces [10,31]. Furthermore, oxygen plasma treatment is a safe and environmentally friendly process (minimal consumption of gas, no reagents, no toxic gases, etc.) which can generate uniform changes throughout the treated samples [31]. Different treatment times were selected to assess the modifications introduced by oxygen plasma treatments, while for alkali treatments, the NaOH solution concentration was varied. In addition, the use of organic and inorganic acids as a final washing step after alkali treatment was evaluated. Few examples can be cited from the literature comprising an experimental assessment of different treatment methods with varying conditions applied to PLA surfaces, including films [32] and composite samples [33], but no references were found regarding 3D structures. In addition, there is a lack of information in the literature about the comparison of how the surface modifications on PLA surfaces evolve over time. These data would be essential if the induced modifications are intended to be used for coating the structures or if storage before utilization is needed (as it is for commercially availability).

The characterization of the surface-treated PLA scaffolds included the assessment on the incorporation of carboxyl groups, the evaluation of the hydrophilicity by measuring the water contact angle, and the analysis of the surface’s chemical composition by energy-dispersive X-ray spectroscopy. Also, the effect of the treatments on the degree of crystallinity and calorimetric properties of the PLA samples was studied by differential scanning calorimetry analysis. Two weeks after applying the treatments, the samples were tested again to evaluate the aforementioned loss of modifications over time. Finally, an enzymatic degradation test using proteinase K enzymes was carried out to assess the degradation rate of the structures, the pH, and the conductivity profile of the media during the five-day test and the morphological and mechanical properties of the surface-treated scaffolds after degradation.

## 2. Materials and Methods

### 2.1. Materials

PLA L105 was purchased from Corbion Purac (Diemen, The Netherlands). This material, supplied in powder form, has a melt flow index of 65 g/10 min and a molecular weight of approximately 105,000 g/mol. The reagents used in this study include sodium hydroxide (NaOH; 30620, Honeywell Fluka^™^, Mt Laurel, NJ, USA), hydrochloric acid (HCl; 131020, Panreac AppliChem, Darmstadt, Germany), citric acid (20282, VWR Chemicals, Fontenay-sous-Bois, France), and acetic acid (ACAC-GIA-2K5, Labkem, Barcelona, Spain).

### 2.2. Manufacturing of Scaffolds

PLA scaffolds were manufactured using a material extrusion process (ISO/ASTM 52900:2015), commonly known as fused deposition modelling (FDM). Specifically, a BQ Hephestos 2 3D printer (Madrid, Spain) was used to manufacture scaffolds with 9.8 mm diameter, 7 mm height, rectangular 0/90 pattern, square-shaped pores, and a 50% theoretical porosity. Other printing settings included a nozzle diameter of 0.4 mm, a layer height of 0.3 mm, a speed of extrusion of 40 mm/s, and a printing temperature of 215 °C.

The continuous PLA filaments fed to the 3D-printed (mean diameter of around 1.75 mm) were obtained using a lab prototype extruder consisting of an 8 mm screw, a cylinder with an L/D ratio of 10, and a 1.6 mm diameter nozzle tip. The extrusion of PLA L105 powder was carried out at a rotating speed of 7 rpm, a temperature of 180 °C, and with a final air- and water-cooling stage.

Apart from the PLA scaffolds, flat-surface samples were manufactured by compression molding to better characterize the PLA surface after applying the different treatments. A Collin P 200 P/M press and the following cycle were used: heating up to 190 °C at a heating rate of 20 °C/min; maintaining the temperature at that constant value for 90 s at 10 bars of pressure, and finally cooling at a rate of 20 °C/min while maintaining the pressure applied in the second step.

As the first step of every experiment included in this work, the samples were measured and weighted to ensure that there were no significant differences regarding these parameters between the different groups tested.

### 2.3. Surface Treatment

#### 2.3.1. Oxygen Plasma Treatments

The plasma treatment of the samples was carried out in a low-pressure device (Zepto Diener electronic GmbH, Ebhause, Germany) comprising a high-frequency generator of 40 kHz. At high frequencies, a more uniform, efficient, and almost continuous discharge can be sustained to maintain the plasma state at lower pressures and energy levels in comparison to direct current (DC) discharges [34]. Oxygen was used as carrier gas (Carburos Metálicos SA, Madrid, Spain), containing less than 500 ppb of H_2_O and less than 400 ppb of N_2_. Oxygen plasma is the most common option for the formation of oxygen functionalities by ion implantation onto the polymeric surface, being proved as a useful and effective surface modification method [11]. The oxygen pressure inside the chamber was fixed at 1.8 mbar, and the surface treatment was applied at a power of 30 W for 1 or 10 min. Both sides of the samples were treated. Depending on the treatment time, the plasma-treated group of samples are referred in this text as PLASMA 1 min or PLASMA 10 min.

#### 2.3.2. Alkali Treatments

PLA samples were placed in a 24-well plate (144530, Thermo Scientific™ Nunc™, Waltham, MA, USA) and immersed during 1 h at room temperature in 2 mL of 0.2 N or 1 N NaOH solutions. Then, the samples were rinsed with distillate water, washed with a 0.1 N HCl solution, and rinsed again with distillate water. PLA samples treated with these methods are referred in the text as 0.2 N NaOH and 1 N NaOH.

In a third alkali surface treatment evaluated, the samples were immersed in 2 mL of 0.2 N NaOH solution, rinsed with distillate water, washed with a 0.05 g/L citric acid solution, and finally rinsed again with distillate water. Samples treated with this method are referred in the text as 0.2 N NaOH + citric acid.

### 2.4. Physicochemical Characterization

#### 2.4.1. Evaluation of Carboxyl Groups on the Treated Surface

The relative surface concentration of carboxyl groups was evaluated by using the Toluidine Blue O (TBO) method [35]. This cationic dye binds to carboxyl groups in a 1:1 molar ratio in a basic medium and can later be desorbed with an acid solution [35,36]. Four replicas of each of the treated scaffold’s groups were tested right after applying the different surface treatments. PLA scaffolds were used as control. The samples were placed individually in centrifuge tubes (CFT011150, 15 mL sterile tubes, Jet Biofil, Guangzhou, China) and immersed in 2 mL of a 0.5 mM Toluidine Blue O (T3260, Sigma Aldrich, St. Louis, MO, USA) solution in 0.1 mM NaOH (pH 10). After 20 h, the samples were transferred to a 24-well plate and rinsed thrice with 1 mL of 0.1 mM NaOH solution. Then, the bounded toluidine was desorbed by adding 2 mL of 50% (*v*/*v*) acetic acid solution for 10 min in each well. Finally, the samples were discarded, and the solutions’ absorbance was measured using a BioTek ELx800 reader (Bio Tek Instruments Inc., Winooski, VT, USA) at an excitation wavelength of 595 nm. The 50% (*v*/*v*) acetic acid solution was used as blank of the measurements.

This procedure was also applied to PLA scaffolds that were surface-treated and then stored in a desiccator for two weeks to evaluate whether the modifications introduced are maintained during that period.

#### 2.4.2. Energy-Dispersive X-ray (EDX) Spectroscopy Analysis

The assessment of the chemical composition of the nontreated, plasma-treated, and alkali-treated PLA samples was carried out by a scanning electron microscope (SEM; Hitachi TM 3030 at an acceleration voltage of 15 kV, Hitachi Ltd., Tokyo, Japan) coupled with an EDX detector. Flat-surface samples obtained by compression molding (as detailed in Section 2.2) were used for this test. The main result of the EDX analysis is the oxygen/carbon ratio (O/C), which is an important indicator of the effectiveness of the proposed treatments to incorporate oxygen groups into the PLA surface. Four measurements were obtained per group on the treatment day. The analysis was repeated two weeks later using the same samples and procedure.

#### 2.4.3. Water Contact Angle (WCA) Measurements

The sessile drop method was used to analyze the WCA of the surface-treated samples, and therefore, the effect of the treatments on the hydrophilicity of the base material. As it is recommended to use flat-surface samples for this test, the samples obtained by compression molding described in Section 2.2 were used. The WCA measurements (*n* = 4) were carried out at room temperature using an Ossila WCA measuring device (Ossila Ltd., Sheffield, UK) and the opensource software ImageJ was used to measure the static contact angle of 2 µL distilled water. The WCA was measured every 24 h during 2 weeks after applying the different surface treatments.

#### 2.4.4. Differential Scanning Calorimetry (DSC) Analysis 

Samples extracted from nontreated and treated scaffolds were subjected to DSC analysis in a differential scanning calorimeter DSC 4000 (Perkin Elmer, Waltham, MA, USA). The scaffolds were surface-treated on the same day or two weeks before the analysis. The samples (*n* = 4) were placed in aluminum crucibles and subjected to a heating/cooling/heating cycle from 30–230 °C, with a nitrogen flow of 20 mL/min and heating and cooling rates of 10 °C/min. The calorimetric data obtained include the glass transition temperature, the onset temperature (at which melting process start), the peak melting temperature and the melting enthalpy. Then, the melting enthalpy values were used to calculate the crystallinity of each group of samples according to the following equation:

(1) $${\%X}_{C}=100\cdot \;\frac{{\Delta H}_{f}}{{\Delta H}_{f}^{{}^{\circ}}}$$

where Xc is the degree of crystallinity, ΔH_f_ is the melting enthalpy of the sample and ΔH_f_^°^ the melting enthalpy of 100% crystalline PLA. The value for ΔH_f_^°^ was 93.7 J/g [37].

### 2.5. Enzymatic Degradation Study

An enzymatic degradation test was carried out to evaluate the effect of the oxygen plasma and alkali surface treatments on the degradation rate of the PLA scaffolds. proteinase *K* enzymes from Tritirachium album (30 units per mg of protein, Merck, Darmstadt, Germany) were used to accelerate the degradation study of the PLA samples [38]. The enzymes were diluted in Tris-HCl buffer (pH 8.6, BioReagent, Merck) at a concentration of 0.2 mg/mL. Sodium azide (ReagentPlus^®^, ≥99.5%, Merck) was added at the same concentration to avoid possible bacterial contamination.

Four replicas per group were placed in a nontreated 24-well plate and 2 mL of degradation media were added per well. PLA scaffolds were used as control. The well plate was maintained in an incubator at 37 °C for 5 days. To avoid the denaturation of the enzymes (due to the lactic acid released during the scaffolds degradation) and therefore maintain a high enzymatic activity, the buffer-enzyme solution was replaced daily. The pH (sensIONTM+PH1, ±0.01, HACH) and conductivity (COND7+, ±0.01, Labbox) of the media were measured every day to follow up the evolution of these parameters during the enzymatic degradation process.

The weight loss of the structures after five days was assessed by using an analytical balance (±0.1 mg, A&D Scales Gemini Series, GR-200, Braunschweig, Germany), while the porosity change of the structures was determined with the following equation [39]:

(2) $$\%\mathrm{porosity}=100\cdot \left(1-\frac{\rho_{\mathrm{ap}}}{\rho_{\mathrm{bulk}}}\right)$$

where ρ_ap_ is the apparent density of the structure and ρ_bulk_ is the density of the bulk material. The latter parameter was estimated by measuring the mass and the dimensions of short filaments of material (*n* = 8), giving a result of 1.22 ± 0.03 g/cm^3^. The apparent density was calculated following a similar protocol for the 3D-printed scaffolds.

Finally, the degraded scaffolds were mechanically characterized under compression test in a LIYI (LI-1065, Dongguan Liyi Environmental Technology Co., Ltd., Dongguan, China) testing machine in displacement control mode. Crosshead speed was set at 1 mm/min. The compressive modulus, offset compressive yield strength (2% deviation from linearity), compression strength, and strain at maximum strength were calculated according to ASTM D695-15. Nondegraded PLA scaffolds were used as reference control (RC).

### 2.6. Statistical Analysis

Statistical analysis was performed using MATLAB software (MATLAB and Statistics Toolbox Release 2021a, The MathWorks, Inc., Natick, MA, USA). The data obtained during this study were analyzed by the Kruskal–Wallis test except for those cases where only two groups were compared. In the latter case, the Wilcoxon two-sided rank sum test was used. The significance level was set to * *p* < 0.05, ** *p* < 0.01 and, *** *p* < 0.001, for statistically significant, highly statistically significant, and very highly statistically significant differences, respectively. All figures and tables show the mean values obtained for each group tested. Standard deviations are represented with error bars in the case of figures.

## 3. Results

### 3.1. Physicochemical Characterization

#### 3.1.1. Weight Loss Due to the Application of the Surface Treatments

To assess the effect of the evaluated surface treatments on the PLA scaffold structure, the weight of each group of samples (*n* = 4) was measured before and after treatment. The scaffolds tested had an initial weight of 0.258 ± 0.01 g, with statistically insignificant differences between groups. After the application of the surface treatments, as shown in Table 1, a statistically significant weight loss was obtained for the 1 N NaOH and 0.2 N NaOH + citric acid groups. The weight loss in the case of alkali treatments was related to NaOH concentration, as the weight decreased more for the most concentrated solution. These data evidenced that these treatments are the most aggressive among the ones tested, so it is expected that they affected the bulk properties of the material (as described below for crystallinity). For plasma-treated samples, the effect of the treatment depends on the time of exposure; scaffolds treated for 10 min showed a statistically significant weight loss (*p* < 0.05) compared with that of the ones treated for 1 min, although in both cases the values remain below 1%.

#### 3.1.2. Evaluation of Carboxyl Groups on the Treated Surface

**Figure 1:**
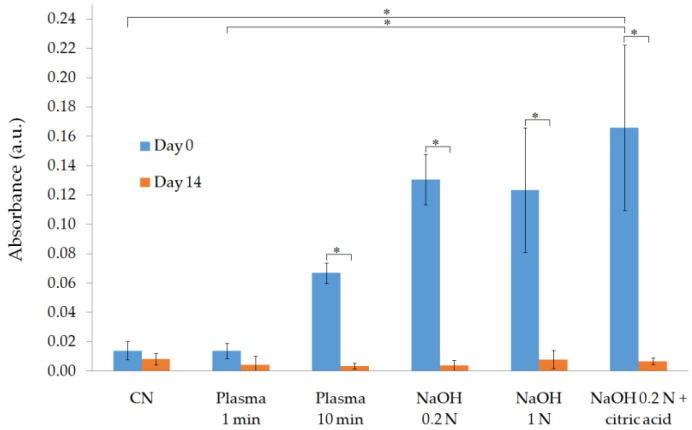
Physicochemical characterization of surface-treated samples by the Toluidine Blue O (TBO) test. * *p* < 0.05.

The incorporation of carboxyl groups into the PLA surface was achieved with the three alkali treatments proposed, as demonstrated by the results obtained at day 0 (day of treatment) with the TBO method (as illustrated in Figure 1).

As expected, the highest mean value of absorbance was obtained for the group of samples treated with 0.2 N NaOH + citric acid (0.17 ± 0.06 a.u.), which showed a significantly higher value of this parameter compared with the samples treated with plasma for 1 min and the nontreated PLA scaffolds used as control. On the other hand, the results suggest that there is no concentration of carboxyl groups on the surface when applying the PLASMA 1 min treatment method, as this is the only group that showed an insignificant difference with the control group at day 0 (absorbance value equal to 0.01 ± 0.01 a.u. in both cases). At day 14, this statement becomes true for all the groups tested.

#### 3.1.3. EDX Analysis

**Figure 2:**
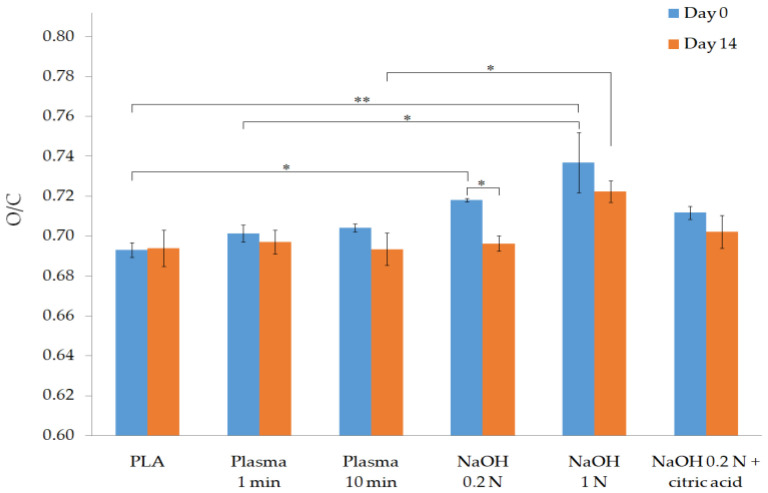
Physicochemical characterization of surface-treated samples by EDX analysis. * *p* < 0.05 and ** *p* < 0.01.

The O/C ratio of each group of samples is represented in Figure 2. In comparison to the PLA control group (O/C ratio equal to 0.693 ± 0.004), statistically significant and highly statistically significant differences were obtained for the 0.2 N NaOH (0.718 ± 0.001) and 1 N NaOH groups (0.737 ± 0.015), respectively. The incorporation of oxygen groups to the PLA surface is especially relevant for 1 N NaOH group, since it showed the highest value at day 0 and is the only group that maintained a significantly higher O/C ratio (0.702 ± 0.008) after 14 days.

#### 3.1.4. WCA Measurements

**Figure 3:**
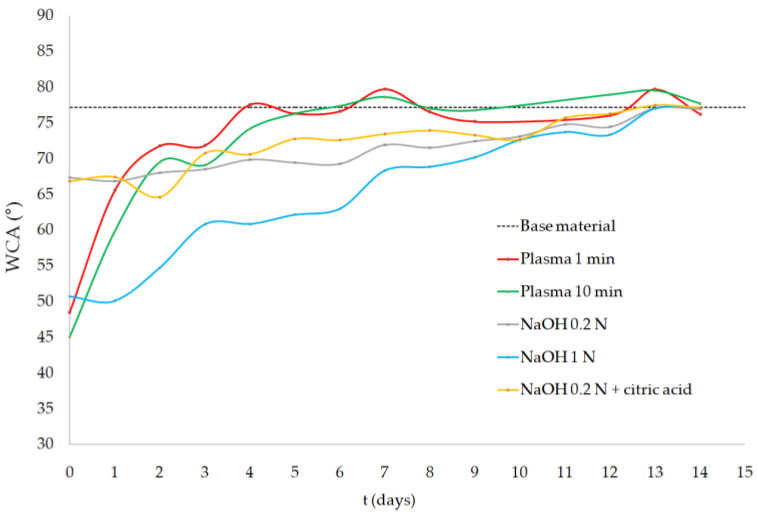
Water contact angle (WCA) measurements of surface-treated samples.

The evolution of the samples’ WCA across two weeks is shown in Figure 3. The mean WCA value of the PLA samples right after treatment (day 0) decreased from the value of 77.2 ± 0.9° (nontreated PLA) to 48.5 ± 3.0° and 45.1 ± 5.2° for the PLASMA 1 min and PLASMA 10 min groups, respectively. The 1 N NaOH group showed a similar result (50.7 ± 3.0°), while the lowest WCA values were obtained for the 0.2 N NaOH (67.4 ± 1.8°) and 0.2 N NaOH + citric acid (66.8 ± 2.7°) treatments. The surface-treated samples tend to recover their initial state but follow a different profile: the effects induced by the oxygen plasma were lost at a higher rate, with the samples treated for 1 min showing a WCA similar to that of the base material in only 4 days (6 days for 10-min plasma); alkali-treated samples, however, reached the WCA value of the base material 13 days after treatment.

#### 3.1.5. Differential Scanning Calorimetry Analysis (DSC)

As shown in Table 2, the proposed surface treatments affect the calorimetric properties of the bulk material. Thus, at day 0, a statistically significant reduction was obtained for the samples treated with 1 N NaOH in terms of glass transition temperature in comparison to the plasma-treated samples. The onset and melting temperatures also tend to be reduced for surface-treated samples. Finally, a significant increase in the melting enthalpy and the crystallinity values were obtained for the 0.2 N NaOH group. This increase in crystallinity, despite being statistically insignificant, was also observed for the PLASMA 10 min and the rest of the alkali treatments.

On the other hand, the differences mentioned at day 0 were not found in the samples treated and analyzed two weeks later. As shown in Table 2b, statistically significant differences were obtained for the value of most of the groups and parameters analyzed compared with day 0, leading to the loss of the modifications introduced by the proposed treatments.

### 3.2. Enzymatic Degradation Study

**Figure 4:**
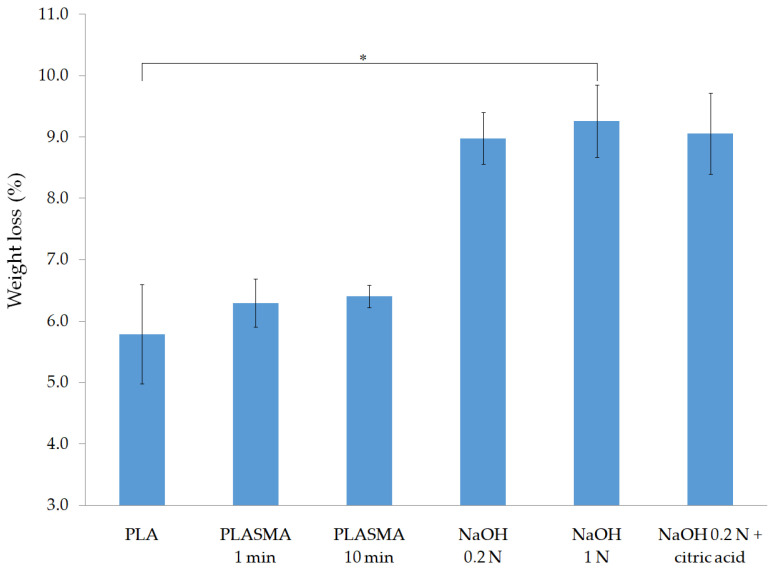
Enzymatic degradation test results of surface-treated samples: % weight loss after five days. * *p* < 0.05.

The weight loss of each group of scaffolds after the five-day enzymatic degradation test is shown in Figure 4. Alkali-treated scaffolds showed a higher level of degradation, with a mean weight loss of around 9% and a statistically significant difference obtained for the 1 N NaOH group compared to that of the nontreated PLA samples.

**Figure 5:**
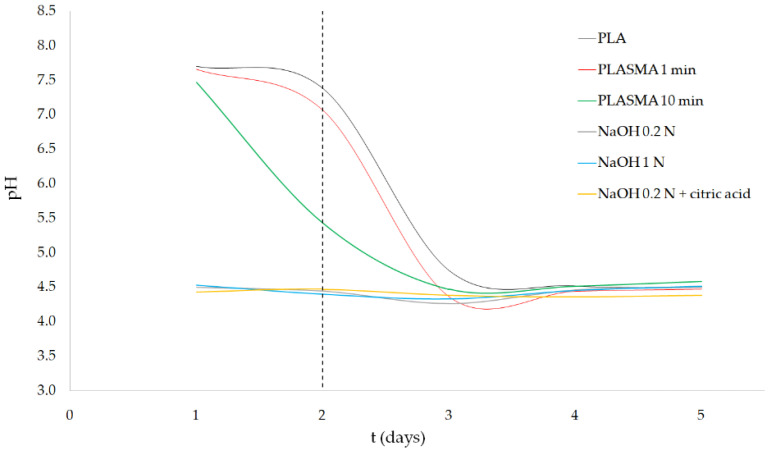
Enzymatic degradation test results of surface-treated samples: pH variation.

Regarding the pH variation of the degradation media (as illustrated in Figure 5), PLASMA 1min samples maintained the initial pH (7.94) almost until day 2, and then rapidly decreased to a value of 4.36 ± 0.01 at day 3. In the case of PLASMA 10 min samples, the pH decreased from day 1, resulting in a less pronounced slope of the curve of pH variation. Alkali-treated groups showed a pH around 4.5 across all 5 days of testing. From day 3, similar results were obtained for all the groups of samples tested.

An increase in the porosity of the scaffolds was also obtained as a consequence of the enzymatic degradation of the structure (as illustrated in Table 3). This increment is statistically significant for the 0.2 N NaOH and 1 N NaOH groups.

Despite being statistically insignificant, the compression test results (as illustrated in Table 3) of the degraded scaffolds showed a reduction in the elastic modulus, compressive yield strength, and compressive strength of the surface-treated scaffolds. The decrease in mechanical properties is more pronounced in the alkali-treated samples, especially for the NaOH 0.2 N + citric acid group, which showed the lowest values for all the parameters evaluated. Notably, the break point of the nondegraded PLA scaffolds, used as reference control (RC), was not reached during the test.

## 4. Discussion

The use of material extrusion techniques offers the possibility of obtaining PLA scaffolds with controlled pore size and porosity so that the characteristics of the 3D construct can be tailored to the patient and target tissue. The precision in the design and manufacturing of the scaffolds is severely compromised when applying an alkali surface treatment, as the weight loss of the samples will surely be accompanied by changes in the struts’ dimensions, microporosity, and mechanical properties of the structure [21]. The percentage of weight loss for scaffolds treated with NaOH depends on the concentration of the solution, being in this study around 2% for 0.2 N NaOH solutions and more than 5% for 1 N NaOH treatment (as illustrated in Table 1). On the other hand, scaffolds treated with plasma showed an insignificant difference of weight before and after treatment for 1 min. When the time of exposure to the oxygen plasma was increased to 10 min, the value of weight loss significantly increased (*p* < 0.05) to 0.21 ± 0.02% compared to that of the group of PLASMA 1 min. Thus, the weight loss depends in this case on the time of treatment, which can be adjusted to maximize the incorporation of oxygen groups while reducing the effects on the structure.

Regarding the physicochemical characterization of the treated samples, the results of the EDX analysis at day 0 revealed that all treatments effectively introduce new oxygen groups on the PLA surface, as the mean O/C ratio increased in comparison to that of the control group in all cases (as illustrated in Figure 2). Alkali treatments showed the highest values of O/C ratio, with significant differences for the 0.2 N NaOH and 1 N NaOH groups compared with the nontreated PLA group. A n insignificant increase of the O/C ratio was obtained when comparing PLA and PLASMA 1 min groups of samples. Similar results were obtained in previous studies that carried out a comparison between oxygen plasma and alkali treatments [32]. Reactive species contained in the oxygen plasma (which include neutral oxygen molecules and atoms, radicals, free electrons, and positively and negatively charged ions [22,40]) are less prone to react with the carbonyl groups of the polymer chain than hydroxide ions, giving the strong nucleophilic nature of the latter [41]. Thus, a greater amount of oxygen groups incorporated into the polymeric surface is expected for alkali treatments.

Results of the TBO test (as illustrated in Figure 1) showed that the 0.2 N NaOH + citric acid group had the highest concentration of carboxyl groups on their surface. This was an expected result, since carboxyl groups are predominantly incorporated when applying an alkali treatment method [32] and the subsequent washing step with an organic acid adds more of these hydrophilic groups onto the PLA surface by ester bond´s hydrolysis [14,18]. Carboxyl groups were also generated, albeit to a lesser extent, by applying the alkali treatments coupled with a washing step using an inorganic acid (HCl) or in the scaffolds treated with the PLASMA 10 min method.

Despite being the only group that showed a nonsignificant difference compared with the control group in the TBO test, the PLASMA 1 min samples showed a reduction of the WCA from 77.2 ± 0.9° to 48.5 ± 3.0° (as illustrated in Figure 3). This highly statistically significant difference (*p* < 0.01) was also observed for the group of PLASMA 10 min, for which the lowest mean WCA value was obtained (45.1 ± 5.2°). These results, coupled with the ones obtained by EDX analysis (as illustrated in Figure 2), suggest that the oxygen groups incorporated to the plasma-treated PLA surface were mainly hydroxyl groups [22,23]. In the case of the alkali treatments, a statistically significant (*p* < 0.05) reduction of the WCA was only obtained for the 1 N NaOH group. The difference in WCA observed between this group and the ones treated with 0.2 N NaOH could be related to the distinct surface roughness generated depending on the solution concentration [15]. Overall, the incorporation of hydroxyl groups and the changes in the surface roughness seem to have a more important effect on the hydrophilicity improvement of the PLA surface than the incorporation of carboxyl groups.

The modifications introduced on the PLA surface were lost after two weeks, as confirmed by the results showed in Figure 1, Figure 2 and Figure 3. Since the WCA was evaluated for 14 days, it was possible to determine the time at which the properties recovered their initial values. The decay on the modifications introduced can be explained by the reorientation and diffusion of the polar groups introduced [30], the rearrangement of hydrophilic and hydrophobic macromolecules fragments within the polymer [28], and the adsorption of ambient humidity [29]; however, samples were kept on a desiccator to limit the latter cause. The rearrangement of the polymer’s chains, which caused the migration of hydrophobic macromolecules fragments to the surface, was evidenced by the recovery of the initial crystallinity value after two weeks (as illustrated in Table 2). In addition, the results suggest that the diffusion or reorientation of the hydroxyl groups introduced is faster than that of the carboxyl groups; samples treated with plasma recovered their initial WCA value in 4–6 days, while for the alkali-treated samples (with a higher formation of carboxyl groups), it took almost two weeks.

The same loss of the modifications introduced by the treatments was observed for the calorimetric properties of the scaffolds (as illustrated in Table 2). At day 0, however, a reduction in glass transition, onset, and peak temperatures was obtained for the treated groups compared with that of the nontreated PLA scaffolds. An increase in the crystallinity of the bulk material was also observed, which could be explained by the increased mobility of the polymer´s chains due to the absorption of water molecules (for alkali treatments) and the incorporation of new oxygen groups into the polymer surface. Both factors affect the movement of the chains in the amorphous region, allowing a rearrangement in part of them to a crystalline structure, according to the principle of thermodynamic equilibrium [29,30]. Another option is the scission of chains in the amorphous regions as a consequence of the surface treatments applied [42]. As previously mentioned, the crystallinity of the base material was recovered within two weeks possibly because hydrophobic macromolecule fragments migrated to the PLA surface [28]. These results give relevant information for the design of procedures regarding scaffold treatment, application of subsequent modifications (such as coating procedures), and storage conditions, thereby having important implications for industrial practice.

Results concerning the enzymatic degradation study showed a significantly higher degradation of the alkali-treated scaffolds after five days (as illustrated in Figure 4), which can be explained by two factors: a) increased surface roughness, expected from the significant weight loss of the scaffolds after treatment (as illustrated in Table 1), and b) higher concentration of carboxyl groups on the scaffold´s surface, which promotes the enzyme–material interaction [43]. Despite the relatively low hydrophilicity of the samples treated with 0.2 N NaOH, according to the WCA measurements (as illustrated in Figure 3), the degradation rate of these groups was as high as that of the 1 N NaOH group. From these results, it can be concluded that the incorporation of carboxyl groups, which are present in the three alkali-treated groups, has a more significant effect on the degradation of the 3D structures than the wettability of the surface.

In Appendix A, the results of a one-day enzymatic degradation test carried out using PLA and 0.2 N NaOH + citric acid groups are presented. While for the alkali-treated group the weight of the scaffolds was reduced by around only 1%, the pH decreased significantly to a value of 4.57 ± 0.01. Similar Ph values were measured every day of the 5-day test (as illustrated in Figure 5) for all three alkali-treated groups, as well as for the plasma-treated groups after day 3. These results suggest that the degradation of the alkali-treated PLA scaffolds progressed to a point where the concentration of dissolved lactic acid was high enough to cause denaturation of the enzymes, stopping the degradation process [44]. This limiting concentration of lactic acid is related to the minimum pH measured during the test.

**Figure 6:**
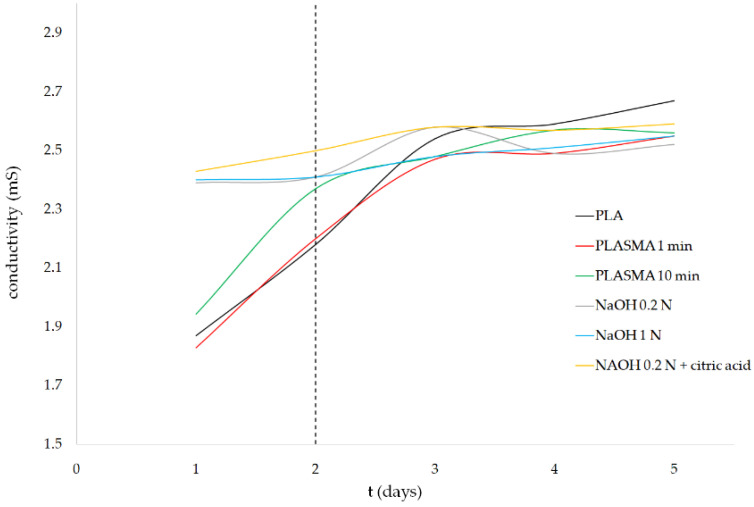
Enzymatic degradation test results of surface-treated samples: conductivity variation.

In contrast to alkali-treated samples, the groups treated with oxygen plasma maintained a higher level of pH during the first two days of the experiment, which could be related to the limited presence of carboxyl groups on their surface. The TBO test (as illustrated in Figure 1) showed that the application of the plasma treatment for 10 min allowed for the incorporation of carboxyl groups, while an insignificant difference was obtained for the PLASMA 1 min group compared with that of the control. Carboxyl groups incorporated into the PLASMA 10 min group enhanced the interaction of the enzymes with the polymer [43] from the beginning of the test, which led to a less drastic decrease in the pH between days 2 and 3 compared with that of the profile observed for the PLASMA 1 min group. In the case of PLA and PLASMA 1 min groups, the enzymatic degradation progressed by a surface-erosion mechanism [45] that enhances the surface roughness with a minimal weight reduction during the first days of the test (similar to the results shown for nontreated PLA scaffolds in Appendix A). From this point, the degradation of the structure is accelerated as new regions of the polymer are exposed to the enzymes due to the increased surface roughness and chains’ cleavage reactions [45]. According to these assumptions, the differences observed in terms of weight loss for the different groups of samples tested (as illustrated in Figure 4) can be related to the first two days of the experiment. The increase of conductivity (for plasma-treated samples) followed by the maintenance of a value around 2.55 mS for all groups tested after day 3 (as illustrated in Figure 6) supported the latter conclusions.

The abrupt release of acidic degradation byproducts could generate a strong inflammatory response, hindering cell growth and affecting the surrounding tissues [46]. Therefore, the degradation profile of PLA scaffolds must be precisely adjusted by optimizing the surface treatment conditions and/or combining the polymeric matrix with ceramic additives that can act as buffers [47,48]. The later strategy could be applied to develop biodegradable 3D structures that gradually release different ceramic particles to counteract the pH decrease of the surrounding environment over time, based on the concept of 4D printing of scaffolds [49,50]. The objective in the addition of ceramic or natural biomaterials to the PLA structure can also be related to the need to ensure enough mechanical support during new tissue formation. As shown in Table 3, a reduction of mechanical properties takes place as the degradation of the PLA scaffolds progresses. Finally, the treated surface of the PLA scaffolds must promote cell adhesion and growth to allow for tissue regeneration. This requirement can be fulfilled by the methods proposed in this study, mainly with the incorporation of carboxylic groups in the case of alkali treatments [19] and the decrease in the inherent hydrophobicity of the base material to values suitable for cell attachment [51] by applying a plasma treatment.

## 5. Conclusions

The present study involves a comparative experimental study between alkali and plasma treatments applied to PLA scaffolds manufactured by AM. The results obtained suggest an important contribution by the carboxyl groups incorporated to the base material surface on the degradation profile of the 3D structures, but not on the hydrophilicity improvement, which was concluded to be more related to the incorporation of hydroxyl groups. The evaluation of these properties over time showed a recovery of the initial state of the surface within two weeks. These findings are of the utmost importance when defining the treatment procedure of the PLA scaffold for biomedical applications, especially if the proposed methods are transferred to the clinic. According to the results, the scaffolds tested possess suitable properties to be further evaluated for biomedical applications.

## Figures and Tables

**Table 1:** Weight loss (%) due to the application of the different surface treatments evaluated.

Group of Samples	%Weight Loss
PLASMA 1 min	0.01 ± 0.02
PLASMA 10 min	0.21 ± 0.02
0.2 N NaOH	1.93 ± 0.11
1 N NaOH	5.85 ± 0.25 ^1^
0.2 N NaOH + citric acid	2.08 ± 0.12 ^2^

^1^ *p* < 0.05 compared with PLASMA 10 min and *p* < 0.01 compared with PLASMA 1 min. ^2^ *p* < 0.05 compared with PLASMA 1 min.

**Table 2:** DSC results of all groups of samples on day of treatment (a) and two weeks later (b).

**(a)**					
**Group of Samples**	**Tg (°C)**	**T_onset_ (°C)**	**T_peak_ (°C)**	**ΔH (J/g)**	**%Xc**
PLA	63.5 ± 1.2	167.0 ± 3.2	176.0 ± 0.1	47.0 ± 1.5	50.1 ± 1.6
PLASMA 1 min	64.2 ± 0.6	169.0 ± 0.1	175.6 ± 0.2	46.8 ± 1.5	50.0 ± 1.6
PLASMA 10 min	64.4 ± 0.5	169.3 ± 0.2	175.6 ± 0.1 ^2^	49.5 ± 1.1	52.9 ± 1.2
0.2 N NaOH	61.5 ± 0.6	169.7 ± 1.2	175.9 ± 0.1	50.5 ± 1.4 ^3^	53.9 ± 2.4 ^4^
1 N NaOH	60.3 ± 0.6 ^1^	169.5 ± 1.2	175.7 ± 0.1	50.3 ± 1.4	53.6 ± 2.7
0.2 NaOH + citric acid	63.4 ± 0.6	164.8 ± 1.2	175.7 ± 0.1	48.8 ± 1.4	52.1 ± 2.1
**(b)**					
**Group of Samples**	**Tg (°C)**	**T_onset_ (°C)**	**T_peak_ (°C)**	**ΔH (J/g)**	**%Xc**
PLA	63.5 ± 1.2	167.0 ± 3.2	176.0 ± 0.1	47.0 ± 1.5	50.1 ± 1.6
PLASMA 1 min	63.9 ± 0.2	169.2 ± 0.2	176.3 ± 0.1 *	47.1 ± 3.0	49.6 ± 2.1
PLASMA 10 min	63.8 ± 0.4	169.3 ± 0.3	176.0 ± 0.1 *	47.8 ± 2.9	51.0 ± 3.1
0.2 N NaOH	63.4 ± 0.4 *	167.8 ± 2.3	176.1 ± 0.1 *	50.9 ± 0.9	54.3 ± 0.9
1 N NaOH	63.2 ± 0.9 *	168.0 ± 2.4	176.0 ± 0.1 *	46.4 ± 2.0 *	49.6 ± 2.1
0.2 NaOH + citric acid	63.5 ± 0.2	166.0 ± 2.3	175.8 ± 0.1 ^1^	49.2 ± 2.8	52.5 ± 3.0

(**a**) ^1^ *p* < 0.01 compared with PLASMA 10 min and *p* < 0.05 compared with PLASMA 1 min. ^2^ *p* < 0.05 compared with PLA. ^3^ *p* < 0.05 compared with PLASMA 1 min. ^4^ *p* < 0.05 compared with PLA and PLASMA 1 min. (**b**) * *p* < 0.05 compared with result at day 0. ^1^ *p* < 0.01 compared with PLASMA 1 min.

**Table 3:** Porosity values (before and after the degradation test) and compression test results.

Group of Samples	Initial Porosity (%)	Final Porosity (%)	Elastic Modulus (MPa)	Compressive Yield Strength (MPa)	Compression Strength (MPa)	Strain at Maximum Strength
RC (compression test)			83.6 ± 7.9	7.2 ± 1.0	-	-
PLA	55.5 ± 2.9	58.0 ± 3.0	81.0 ± 10.7	7.0 ± 1.5	9.7 ± 2.0	0.24 ± 0.07
PLASMA 1 min	56.0 ± 2.5	59.0 ± 2.1	72.7 ± 4.9	6.5 ± 1.7	9.9 ± 2.9	0.27 ± 0.04
PLASMA 10 min	56.1 ± 1.6	58.8 ± 1.5	73.7 ± 13.3	6.6 ± 1.4	9.2 ± 1.9	0.24 ± 0.03
NaOH 0.2 N	55.9 ± 1.8	60.1 ± 1.7 *	69.2 ± 13.5	6.9 ± 1.5	8.9 ± 0.9	0.23 ± 0.04
NaOH 1 N	58.8 ± 0.9	61.7 ± 1.6 *	67.7 ± 8.4	6.0 ± 0.8	8.4 ± 1.1	0.25 ± 0.01
NaOH 0.2 N + citric acid	56.4 ± 2.4	60.8 ± 2.3	63.2 ± 7.5	5.2 ± 0.8	8.2 ± 1.8	0.26 ± 0.02

* *p* < 0.05 compared with initial porosity of this group.

## Data Availability

The data presented in this study are available on request from the corresponding author.

## Appendix A

An enzymatic degradation test was carried out using PLA scaffolds treated with 0.2 N NaOH and washed with citric acid for one day, following the procedure described in Section 2.5. PLA scaffolds were used as control. The results of this test are shown in Table A1, including weight loss of the samples, pH and conductivity of the media, and porosity change after degradation.

**Table A1:** Results of the one-day enzymatic degradation test.

Group of Samples	Weight Loss (%)	pH	Conductivity (mS)	Initial Porosity (%)	Final Porosity (%)
PLA	0.01 ± 0.01	7.56 ± 0.01	2.00 ± 0.01	44.1 ± 3.8	43.1 ± 2.2
NaOH 0.2 N + citric acid	1.37 ± 0.11 ^1^	4.57 ± 0.01 ^1^	2.41 ± 0.01 ^1^	41.0 ± 1.3	44.2 ± 2.8

^1^ *p* < 0.05 compared with PLA.

## References

[1] Puppi D., Chiellini F., Piras A.M., Chiellini E. (2010). Polymeric materials for bone and cartilage repair. Prog. Polym. Sci..

[2] Wang X., Xu S., Zhou S., Xu W., Leary M., Choong P., Qian M., Brandt M., Xie Y.M. (2016). Topological design and additive manufacturing of porous metals for bone scaffolds and orthopaedic implants: A review. Biomaterials.

[3] Vallet-Regí M., Ruiz-Hernández E. (2011). Bioceramics: From bone regeneration to cancer nanomedicine. Adv. Mater..

[4] Ulery B.D., Nair L.S., Laurencin C.T. (2011). Biomedical applications of biodegradable polymers. J. Polym. Sci. Part B Polym. Phys..

[5] Liu X., Ma P.X. (2004). Polymeric scaffolds for bone tissue engineering. Ann. Biomed. Eng..

[6] Foresti R., MacAluso C., Rossi S., Selleri S., Perini P., Freyrie A., Raposio E., Fenaroli P., Concari G., De Filippo M. 3D reconstruction cutting and smart devices for personalized medicine. Proceedings of the 2020 Italian Conference on Optics and Photonics.

[7] Foresti R., Ghezzi B., Vettori M., Bergonzi L., Attolino S., Rossi S., Tarabella G., Vurro D., von Zeppelin D., Iannotta S. (2021). 3D printed masks for powders and viruses safety protection using food grade polymers: Empirical tests. Polymers.

[8] Germain L., Fuentes C.A., van Vuure A.W., des Rieux A., Dupont-Gillain C. (2018). 3D-printed biodegradable gyroid scaffolds for tissue engineering applications. Mater. Des..

[9] Zhao H., Li L., Ding S., Liu C., Ai J. (2018). Effect of porous structure and pore size on mechanical strength of 3D-printed comby scaffolds. Mater. Lett..

[10] Richbourg N.R., Peppas N.A., Sikavitsas V.I. (2019). Tuning the biomimetic behavior of scaffolds for regenerative medicine through surface modifications. J. Tissue Eng. Regen. Med..

[11] Chu P.K., Chen J.Y., Wang L.P., Huang N. (2002). Plasma-surface modification of biomaterials. Mater. Sci. Eng. R Rep..

[12] Croll T.I., O’Connor A.J., Stevens G.W., Cooper-White J.J. (2004). Controllable surface modification of poly(lactic-co-glycolic acid) (PLGA) by hydrolysis or aminolysis I: Physical, chemical, and theoretical aspects. Biomacromolecules.

[13] De Jong S.J., Arias E.R., Rijkers D.T.S., Van Nostrum C.F., Kettenes-Van Den Bosch J.J., Hennink W.E. (2001). New insights into the hydrolytic degradation of poly(lactic acid): Participation of the alcohol terminus. Polymers.

[14] Guo C., Xiang M., Dong Y. (2015). Surface modification of poly (lactic acid) with an improved alkali-acid hydrolysis method. Mater. Lett..

[15] Nam Y.S., Yoon J.J., Lee J.G., Park T.G. (1999). Adhesion behaviours of hepatocytes cultured onto biodegradable polymer surface modified by alkali hydrolysis process. J. Biomater. Sci. Polym. Ed..

[16] Shao J., Chen S., Du C. (2015). Citric acid modification of PLLA nano-fibrous scaffolds to enhance cellular adhesion, proliferation and osteogenic differentiation. J. Mater. Chem. B.

[17] Lao L., Tan H., Wang Y., Gao C. (2008). Chitosan modified poly(l-lactide) microspheres as cell microcarriers for cartilage tissue engineering. Colloids Surf. B Biointerfaces.

[18] Martin V., Ribeiro I.A., Alves M.M., Gonçalves L., Claudio R.A., Grenho L., Fernandes M.H., Gomes P., Santos C.F., Bettencourt A.F. (2019). Engineering a multifunctional 3D-printed PLA-collagen-minocycline-nanoHydroxyapatite scaffold with combined antimicrobial and osteogenic effects for bone regeneration. Mater. Sci. Eng. C.

[19] Arima Y., Iwata H. (2015). Preferential adsorption of cell adhesive proteins from complex media on self-assembled monolayers and its effect on subsequent cell adhesion. Acta Biomater..

[20] Li B., Ma Y., Wang S., Moran P.M. (2005). Influence of carboxyl group density on neuron cell attachment and differentiation behavior: Gradient-guided neurite outgrowth. Biomaterials.

[21] Chen W., Nichols L., Brinkley F., Bohna K., Tian W., Priddy M.W., Priddy L.B. (2021). Alkali treatment facilitates functional nano-hydroxyapatite coating of 3D printed polylactic acid scaffolds. Mater. Sci. Eng. C.

[22] Scaffaro R., Lopresti F., Sutera A., Botta L., Fontana R.M., Gallo G. (2017). Plasma modified PLA electrospun membranes for actinorhodin production intensification in *Streptomyces coelicolor* immobilized-cell cultivations. Colloids Surf. B Biointerfaces.

[23] Nakagawa M., Teraoka F., Fujimoto S., Hamada Y., Kibayashi H., Takahashi J. (2006). Improvement of cell adhesion on poly(L-lactide) by atmospheric plasma treatment. J. Biomed. Mater. Res. Part A.

[24] Jordá-Vilaplana A., Fombuena V., García-García D., Samper M.D., Sánchez-Nácher L. (2014). Surface modification of polylactic acid (PLA) by air atmospheric plasma treatment. Eur. Polym. J..

[25] Wang M., Favi P., Cheng X., Golshan N.H., Ziemer K.S., Keidar M., Webster T.J. (2016). Cold atmospheric plasma (CAP) surface nanomodified 3D printed polylactic acid (PLA) scaffolds for bone regeneration. Acta Biomaterialia.

[26] Yamaguchi M., Shinbo T., Kanamori T., Wang P.C., Niwa M., Kawakami H., Nagaoka S., Hirakawa K., Kamiya M. (2004). Surface modification of poly(L-lactic acid) affects initial cell attachment, cell morphology, and cell growth. J. Artif. Organs.

[27] Teixeira B.N., Aprile P., Mendonça R.H., Kelly D.J., da Silva Moreira Thiré R.M. (2019). Evaluation of bone marrow stem cell response to PLA scaffolds manufactured by 3D printing and coated with polydopamine and type I collagen. J. Biomed. Mater. Res. Part B Appl. Biomater..

[28] Moraczewski K., Stepczyńska M., Malinowski R., Rytlewski P., Jagodziński B., Zenkiewicz M. (2016). Stability studies of plasma modification effects of polylactide and polycaprolactone surface layers. Appl. Surf. Sci..

[29] Li J., Oh K., Yu H. (2005). Surface rearrangements of oxygen plasma treated polystyrene: Surface dynamics and humidity effect. Chin. J. Polym. Sci..

[30] Canal C., Molina R., Bertran E., Erra P. (2004). Wettability, ageing and recovery process of plasma-treated polyamide 6. J. Adhes. Sci. Technol..

[31] Cheng Y.L., Wang Y.K., Chen P., Deng S.B., Ruan R. (2014). Non-thermal plasma assisted polymer surface modification and synthesis: A review. Int. J. Agric. Biol. Eng..

[32] Durán I.R., Vanslambrouck S., Chevallier P., Hoesli C.A., Laroche G. (2020). Atmospheric pressure cold plasma versus wet-chemical surface treatments for carboxyl functionalization of polylactic acid: A first step toward covalent immobilization of bioactive molecules. Colloids Surf. B Biointerfaces.

[33] Gibeop N., Lee D.W., Prasad C.V., Toru F., Kim B.S., Song J. (2013). Il Effect of plasma treatment on mechanical properties of jute fiber/poly (lactic acid) biodegradable composites. Adv. Compos. Mater..

[34] Denes F.S., Manolache S. (2004). Macromolecular plasma-chemistry: An emerging field of polymer science. Prog. Polym. Sci..

[35] Uchida E., Uyama Y., Ikada Y. (1993). Sorption of low-molecular-weight anions into thin polycation layers grafted onto a film. Langmuir.

[36] Liu Y., He T., Gao C. (2005). Surface modification of poly(ethylene terephthalate) via hydrolysis and layer-by-layer assembly of chitosan and chondroitin sulfate to construct cytocompatible layer for human endothelial cells. Colloids Surf. B Biointerfaces.

[37] Garlotta D. (2001). A literature review of poly(lactic acid). J. Polym. Environ..

[38] Tsuji H., Muramatsu H. (2001). Blends of aliphatic polyesters: V. Non-enzymatic and enzymatic hydrolysis of blends from hydrophobic poly(L-lactide) and hydrophilic poly(vinyl alcohol). Polym. Degrad. Stab..

[39] Yang G.H., Kim M., Kim G. (2017). Additive-manufactured polycaprolactone scaffold consisting of innovatively designed microsized spiral struts for hard tissue regeneration. Biofabrication.

[40] Vesel A., Mozetic M. (2017). New developments in surface functionalization of polymers using controlled plasma treatments. J. Phys. D Appl. Phys..

[41] Ouellette R.J., Rawn J.D. (2015). Nucleophilic Substitution and Elimination Reactions. Organic Chemistry Study Guide.

[42] Yip J., Chan K., Sin K.M., Lau K.S. (2004). Comprehensive study of polymer fiber surface modifications Part 2: Low-temperature oxygen-plasma treatment. Polym. Int..

[43] Ye P., Wan R.B., Wang X.P. (2009). Quantitative enzyme immobilization: Control of the carboxyl group density on support surface. J. Mol. Catal. B Enzym..

[44] Hegyesi N., Zhang Y., Kohári A., Polyák P., Sui X., Pukánszky B. (2019). Enzymatic degradation of PLA/cellulose nanocrystal composites. Ind. Crop. Prod..

[45] Tsuji H., Ishida T. (2002). Poly(l-lactide). X. Enhanced surface hydrophilicity and chain-scission mechanisms of poly(l-lactide) film in enzymatic, alkaline, and phosphate-buffered solutions. J. Appl. Polym. Sci..

[46] Araque-Monrós M.C., Vidaurre A., Gil-Santos L., Gironés Bernabé S., Monleón-Pradas M., Más-Estellés J. (2013). Study of the degradation of a new PLA braided biomaterial in buffer phosphate saline, basic and acid media, intended for the regeneration of tendons and ligaments. Polym. Degrad. Stab..

[47] Schiller C., Epple M. (2003). Carbonated calcium phosphates are suitable pH-stabilising fillers for biodegradable polyesters. Biomaterials.

[48] Niaza K.V., Senatov F.S., Kaloshkin S.D., Maksimkin A.V., Chukov D.I. (2016). 3D-printed scaffolds based on PLA/HA nanocomposites for trabecular bone reconstruction. J. Phys. Conf. Ser..

[49] Rafiee M., Farahani R.D., Therriault D. (2020). Multi-Material 3D and 4D Printing: A Survey. Adv. Sci..

[50] Foresti R., Rossi S., Selleri S. Bio composite materials: Nano functionalization of 4D bio engineered scaffold. Proceedings of the 2019 IEEE International Conference on BioPhotonics.

[51] Chen S., Guo Y., Liu R., Wu S., Fang J., Huang B., Li Z., Chen Z., Chen Z. (2018). Tuning surface properties of bone biomaterials to manipulate osteoblastic cell adhesion and the signaling pathways for the enhancement of early osseointegration. Colloids Surf. B Biointerfaces.

